# Video Q&A: Allergies and allergen immunotherapy - an interview with Alfred William Frankland

**DOI:** 10.1186/1741-7015-12-11

**Published:** 2014-01-21

**Authors:** A William Frankland

**Affiliations:** 1 London Allergy Clinic, New Cavendish Street, London, UK

## Abstract

In this video Q&A, we talk to Dr Alfred William Frankland about the highlights of his career, including working alongside Sir Alexander Fleming, co-founding the British Allergy Society, and introducing pollen counts to UK weather forecasts. We also discuss his opinions on why misconceptions about allergies and allergen immunotherapy still exist.

Please see related article: http://www.biomedcentral.com/1741-7015/11/255.

## Dr Alfred William Frankland talks about allergies and allergen immunotherapy.

## Introduction

Dr Alfred William Frankland, also referred to as “the grandfather of allergy”, has made extraordinary contributions to medicine over the last 60 years. He pioneered the very first double-blind randomized controlled trial on allergen immunotherapy, and his research into pollen allergies led to the inclusion of pollen counts into the UK weather forecasts in 1963. At 101, he remains very active as he continues to practice as a physician, attends international conferences and continues to publish scientific papers.

In this interview, we talk to Dr Frankland about the highlights of his career, about his experiences of working alongside Sir Alexander Fleming, and ask for his opinion about why some misconceptions about allergies and allergen immunotherapy still exist today.

## Edited transcript

### 1. Can you start by describing your early life and what inspired you to enter medicine?

Why did I take up medicine? I think because when I was young I rather despised a GP. This was in the Lake District, in Cumbria. He came to see us when I had bovine tuberculosis and was kept in bed for four months. They said it was all due to my tonsils, which were then removed. Anyhow, I didn’t like this old man and I thought, “*When I grow up, I’d like to be a doctor*.” That’s really how I started, I suppose.

When I was a medical student, as part of my training, I had to make two visits to the eye department and to the ear, nose and throat department. When I went to the eye department I saw a blonde girl with beautiful eyes and thought, “*I must get to know this girl*.” When I went there a second time I was sure about it.

So what happened? They didn’t have what was called in those days a house physician or a house surgeon - so they employed a senior medical student. To get to know this girl in the eye outpatients (she was working as an orthoptist), I applied for this job. It was a risky thing to do because you were kept very, very busy. You put on a long white coat, everyone called you ‘Doctor’ and you acted as a doctor for six months - even though you were still a medical student. Anyhow, I got to know the girl, and we were finally married in 1941. I’m a slow worker!

### 2. You joined St Mary’s Hospital in 1934 and qualified in 1938. By 1946, you found yourself becoming interested in allergy - how did that come about?

I became interested in allergy completely by chance. After six and a half years in the war (three and a half years as a prisoner of war), I went back to my own teaching hospital - St. Mary’s. We were given what was called “ex-service registrar” jobs. I’d chosen to do dermatology - special clinic. I hadn’t been doing this for more than a month when a notice went up saying they wanted a doctor in the allergy department for two mornings a week and one afternoon. I was free on those, so here was a chance of getting a little more expertise in a subject which at that time I knew nothing about. So I started in the allergy department and after six weeks liked it so much I went to Dr Freeman, my chief, and said, *”Could I be full time?”* Since that time, I’ve been a full time allergist.

### 3. So while you were at St Mary’s, you worked with Dr John Freeman who was working on allergy, and also with Sir Alexander Fleming who was working on penicillin. It’s been said that as you were on good terms with both of them, you acted as a bridge between their research. Can you describe that time for us?

Well, Dr. Freeman - we always called him ‘JF’ and Fleming, we called him ‘Flem’ - didn’t get on at all well together. My chief was Freeman but after a short time I was looking after what was called the “experimental ward”. Therefore, I had to see Professor Fleming every morning at 10 o’clock to talk about the patients. But we never talked about them as he wasn’t interested in clinical medicine. He was a fascinating man in so many ways, and extremely clever. I really enjoyed these 10 o’clock meetings. It happened that he didn’t get on at all well with my real chief, Freeman. But that didn’t matter - I just continued, as it were, having two chiefs.

After three or four years I never saw anything of Freeman, but I inherited all his expertise. Including, of course, the largest pollen farm in the world. We produced pollen not only for experimental purposes, but we also sold pollen to America and Spain and so on. The pollen came, for the most part, from Timothy grass. I loved going down to the farm to see how to collect pollen, and when I first went there the farm was run by Dorothy Noon, who was the sister of Leonard Noon (who gave the first successful treatment of seasonal hay fever at St Mary’s Hospital in 1911). So, an allergy clinic has been running at St. Mary’s from about 1906 till the present day - and that’s a very long time!

### 4. So since the regular use of penicillin as an antibiotic, you started to see some patients developing allergic reactions. At the time, the concept of allergy was still very new, and particularly the concept of having an allergic reaction to drugs was very new. How did you deal with this at the time?

In those days, we had to recognize that this new wonder drug, Penicillin, was changing the whole of medicine by curing diseases that had been killing people. But, like all drugs to this present day, it could cause side-effects. The side-effects, as far as the allergy was concerned, were allergic reactions. In those days, and almost still today - it was very difficult to work out exactly what bit of the penicillin molecule people are allergic to. We just said this was an allergic reaction to penicillin and you mustn’t have any more penicillin because, instead of being beneficial, it could be fatal.

The sequel of all that, of course, was the book that Fleming had written. This was a multiple authorship book just after the war. Every doctor in the country had to have this bible of how to treat all sorts of infectious diseases and so on. He sold, in fact, 175,000 copies of this book. Which is a little unusual for a medical book, but it was the bible of treatment of infection.

After two or three years, Butterworths (the publishers of the book) decided that it must have a second edition, for two reasons. They wanted two more chapters: one on the new mycin that had just been discovered by Selman Waksman (streptomycin), and another chapter on allergic reactions. Fleming was always against this idea. In fact, he always insisted that reactions to penicillin (in those days a lot of it was given by injections) was due to impurities in the preparation. But after six months, the publisher finally said, “*You must get this second edition out!”* So, one morning when I went to see Fleming he said to me: “*Frankland, you’re going to write this chapter on penicillin reactions. I’ll give you a week to do it; 3,000 words - not more; and not more than 30 references.”*

So I wrote it that week. On Monday morning (I still remember giving it to him) he said, *“I will read it tonight and I’ll tell you tomorrow morning what I think about it.”* So, on Tuesday morning I was really quite dreading what he might say. But no, he was as always his very nice self and said, “*I’m not saying that I agree with everything that you have written, but I’m not going to change it, except your very last sentence on the last page.”*

I had written: “*With the increasing use of penicillin, it is to be expected that allergic reactions will become more common.”* He crossed this last sentence out and wrote in his beautiful handwriting “*With increasing use of penicillin, reactions due to impurities will become less common.”* And that’s what’s in the book. To some extent, he was right - but I was also right!

### 5. When you co-founded the British Allergy Society in the late 1940s, you and your colleagues - which also included Professor Jack Pepys were told to define allergy. How hard was it to reach a consensus?

We didn’t! We wanted to form this British Allergy Society, and so it was advertised that we were going to have this meeting at St Mary’s. There were going to be two speakers: one was John Freeman, who was going to talk about hay fever and allergic reactions to grass pollen; and the other was Sir Henry Dale, who was going to talk about allergy in general. But Dale was really in charge.

They both gave interesting lectures but at the end of it, Dale said: *“If you’re going to form an Allergy Society, you should agree on some definition of allergy” and* there and then nominated me as the Secretary of this new society*.* His suggestion was that everyone should write their definition of allergy and send it to me by post. About 12 arrived in the next week or so, but I tore them all up as most of them were not what I would call a good definition. One which certainly wasn’t a definition, but I still remember, came from Scotland and said: “*Allergy is a grossly overused word by the lay public.”* That was more than 60 years ago, and it’s still true today.

### 6. Thanks to your work on tracking seasonal variations of pollen counts, levels of pollen became part of mainstream media broadcasting in 1963. This has had a huge impact on public health, and raised public health awareness regarding pollen. How did that make you feel?

It was very interesting. Freeman was very keen on pollen and I wanted to enlarge on his interest. We had these special clinics where we used to see patients who only had seasonal hay fever with or without associated asthma. We saw these vast numbers of people.

In fact this clinic was so popular that at one point the House Governor asked me to explain why there were so many people. He said, *“You are taking over St. Mary’s Hospital with your clinic! Will you tell me how many you’ve seen this spring?”* Two days later, I saw his secretary and I gave him a figure - we had seen just over 6,000 people! Now this is a large amount and people just don’t believe we could see so many people. But we had two complete teams running (I paid medical students to take the history or to do the skin prick test and so on). My proud boast was that no patient… from the time that they arrived to the time that they left, would be there for more than one hour. The first appointment for the clinic was scheduled at two o’clock, but the patients were fully going at half past one because so many people arrived early. So people weren’t kept waiting for long at all. But I was ticked off for having this popular clinic by the House Governor, and told I had to halve the numbers of patients by the next year. So I had to cut it down to just over 3,000.

### 7. Since your seminal paper in 1954, on prophylaxis of summer hay fever and asthma, there have been numerous studies published on allergen immunotherapy for the treatment of allergic respiratory diseases. However, there are still some misconceptions about this type of therapy and also about allergy in particular. You’ve outlined some of this, alongside Professor Moisés Calderón and Professor Pascal Demoly, in a paper we’ve published in *BMC Medicine*. Despite the clinical evidence available, why do you think these misconceptions about allergen immunotherapy still exist today?

There’s so much to learn about what is happening when you’re using immunotherapy. In the 1950s we used to give injections, but I think more and more we’re going to go on to the idea that as nature intended, if you’re going to bring about immune tolerance - it’s going to be given orally. That’s what nature did through breast milk and so on. But so many people distrust whatever you’re doing perhaps from not knowing how immunotherapy works, whether it’s given by injection or given orally. And the answer is - we really don’t know, it’s a very complicated business!

Unfortunately in this country, although some of the best research work in the world is done here - the number of allergists who have to treat patients with allergic diseases is amazingly small. We’ve nothing like enough consultants and clinics to deal with this problem. It’s a fascinating subject to see what’s happening intracellularly and we’re learning a terrific amount and getting new ideas all the time.

There was a double-blind controlled trial that I did at the same time in 1954 on infective asthma. In those days bacteriologists were absolutely in charge. If hay fever or asthma wasn’t allergic, they thought it was infective. Therefore, my chief would take a swab, and if any bacteria were growing, he said, “*Oh, people are reacting to this, they haven’t got an immunity,”* so he made what’s called an autogenous bacterial vaccine.

That was the treatment then, and there were a lot of people receiving this treatment. I did a double-blind controlled trial because I didn’t believe it gave any scientific help. We found that the placebo injections (saline) gave the same amount of help as these very expensive and difficult to produce autogenous bacterial vaccines. Well, 49% were helped by the saline; 51% were helped by the autogenous bacterial vaccines. You don’t require any knowledge of statistics to know that these two figures, in quite a large series of people, are the same.

That paper caused so much interest that in fact I was invited to go and give a talk at the International College of Physicians in Madrid. Two months later, I gave a similar talk in Los Angeles, so it was interesting that trial caused, internationally, much more interest than the double-blind controlled trial on pollen. One of the reasons was that statistics and double-blind controlled trials were not common at the time. The publication of the bacterial vaccine trial showed that a treatment didn’t work - and in those days, if you did anything or used a drug that didn’t work, you didn’t talk about it. Here was I publishing a statistically significant double blind trial on something that didn’t work!

### 8. You’re now 101, yet over the years you’ve faced a few life-threatening situations such as being a prisoner of war for three and a half years in Singapore during WWII, and also going into anaphylactic shock during a set of self-experiments on your allergic reaction to a South American insect. How are you still here?

Yes. People ask me how is it that I have reached 101, and I say the answer to that is: I have been so near death so many times, but I’ve always just missed! That’s why I’m still alive.

### 9. There’s no doubt you’ve led a remarkable life, Dr Frankland, and thanks to your dedication to medicine, people with hay fever benefit from being able to plan for the summer. What would be your advice to physicians - particularly allergists - of today?

Advice to allergists? Well, in this country we are very good at doing research in allergic problems and it’s a very complicated business looking at what the cells are doing. The whole of immunochemistry in all branches of medicine is advancing so much. I always say what’s needed is research, research, research, and a lot of very clever doctors doing the research. The problem is, there are a few professorial places in the UK that run an allergy clinic and do allergy research. Compared with America, or even most countries in Europe, we are not spending enough on research and we haven’t anything like enough clinics going and enough doctors - general practitioners as well as others - who know almost all the elements of this very complicated business of allergy.

### 10. Where can I find out more?

See reference list [1-5].

## Supplementary Material

Dr Alfred William Frankland talks about allergies and allergen immunotherapy Click here for file

## References

[B1] Frankland AW Augustin R Prophylaxis of summer hay-fever and asthma: a controlled trial comparing crude grass-pollen extracts with the isolated main protein component Lancet 1954 266 1055 1057 13164324 10.1016/s0140-6736(54)91620-7

[B2] Frankland AW Hughes WH Gorrill RH Autogenous bacterial vaccines in treatment of asthma BMJ 1955 2 941 944 10.1136/bmj.2.4945.941 13260633 PMC1981089

[B3] Cohen SG Frankland AW Dworetzky M Noon and Freeman on prophylactic inoculation against hay fever J Allergy Clin Immunol 2003 111 1142 1150 10.1016/S0091-6749(03)80153-7 12743591

[B4] Fleming A Penicillin, Its Practical Application 1950 2 London: Butterworth

[B5] Calderón MA Frankland AW Demoly P Allergen immunotherapy and allergic rhinitis: false beliefs BMC Med 2013 11 255 10.1186/1741-7015-11-255 24314210 PMC4029303

